# Interaction of vascular endothelial cells with hydrophilic fullerene nanoarchitectured structures in 2D and 3D environments

**DOI:** 10.1080/14686996.2024.2315014

**Published:** 2024-02-13

**Authors:** Tsai-Yu Chen, Kun-Chih Cheng, Pei-Syuan Yang, Lok Kumar Shrestha, Katsuhiko Ariga, Shan-hui Hsu

**Affiliations:** a Institute of Polymer Science and Engineering, National Taiwan University, Taipei, Taiwan, R.O.C; b Supermolecules Group, Research Center for Materials Nanoarchitectonics (MANA), National Institute for Materials Science (NIMS), Tsukuba, Japan; c Department of Materials Science, Faculty of Pure and Applied Sciences, University of Tsukuba, Tsukuba, Japan; d Graduate School of Frontier Sciences, The University of Tokyo, Kashiwa, Chiba, Japan; e Institute of Cellular and System Medicine, National Health Research Institutes, Miaoli, Taiwan, R.O.C

**Keywords:** Self-assembled fullerenes, nanotube, nanowhisker, self-healing hydrogel, endothelial cells, vascularization

## Abstract

The interaction between diverse nanoarchitectured fullerenes and cells is crucial for biomedical applications. Here, we detailed the preparation of hydrophilic self-assembled fullerenes by the liquid-liquid interfacial precipitation (LLIP) method and hydrophilic coating of the materials as a possible vascularization strategy. The interactions of vascular endothelial cells (ECs) with hydrophilic fullerene nanotubes (FNT-P) and hydrophilic fullerene nanowhiskers (FNW-P) were investigated. The average length and diameter of FNT-P were 16 ± 2 μm and 3.4 ± 0.4 μm (i.e. aspect ratios of 4.6), respectively. The average length and diameter of FNW-P were 65 ± 8 μm and 1.2 ± 0.2 μm (i.e. aspect ratios of 53.9), respectively. For two-dimensional (2D) culture after 7 days, the ECs remained viable and proliferated up to ~ 420% and ~ 400% with FNT-P and FNW-P of 50 μg/mL, respectively. Furthermore, an optimized chitosan-based self-healing hydrogel with a modulus of ~400 Pa was developed and used to incorporate self-assembled fullerenes as *in vitro* three-dimensional (3D) platforms to investigate the impact of FNT-P and FNW-P on ECs within a 3D environment. The addition of FNW-P or FNT-P (50 μg/mL) in the hydrogel system led to proliferation rates of ECs up to ~323% and ~280%, respectively, after 7 days of culture. The ECs in FNW-P hydrogel displayed an elongated shape with aligned morphology, while those in FNT-P hydrogel exhibited a rounded and clustered distribution. Vascular-related gene expressions of ECs were significantly upregulated through interactions with these fullerenes. Thus, the combined use of different nanoarchitectured self-assembled fullerenes and self-healing hydrogels may offer environmental cues influencing EC development in a 3D biomimetic microenvironment, holding promise for advancing vascularization strategy in tissue engineering.

## IMPACT STATEMENT

Self-assembled fullerenes with large aspect ratios modulate the morphology and gene expression of endothelial cells within a soft biomimetic 3D microenvironment, representing a promising new vascularization strategy in tissue engineering.

## GRAPHICAL ABSTRACT

**Figure:**
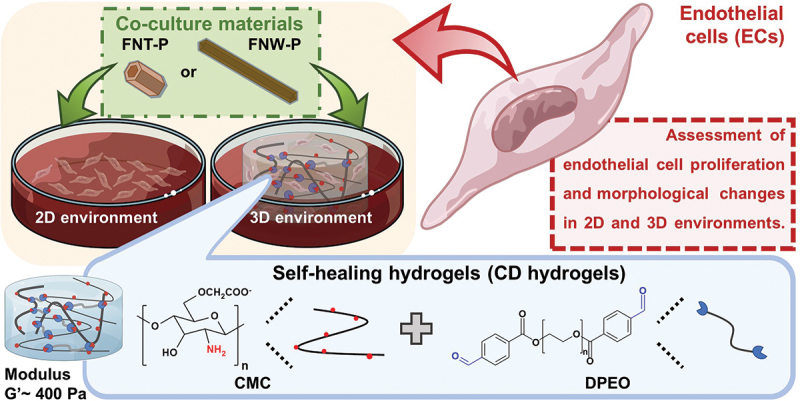

## 1. Introduction

Nanocarbons, serving as pioneering nanomaterials in material science research, have broad applications in our daily life, including life science, energy, and the environment [1]. Nanocarbon family such as carbon nanotubes, fullerenes, and graphene are celebrated for unique properties in terms of chemistry, photoelectricity, thermal conductivity, and mechanical strength [2]. Among these nanocarbons, zero-dimensional fullerene molecules possess high self-assemble capabilities to create a variety of higher-dimensional structures through different nanoarchitectonic processes [3]. In fullerene nanoarchitectonics, various methods are employed to fabricate assembled materials, including template synthesis, slow evaporation of fullerene solutions, vapor deposition, and liquid-liquid interfacial precipitation (LLIP) [4]. Hydrophobic fullerenes can self-assemble into diverse nanoarchitectured structures, such as nanowhiskers, nanotubes, nanorods, nanosheets, and micro-cubes, through the LLIP method [5,6]. Fullerene nanowhiskers (FNWs) consist of slender crystalline fibers and find diverse applications, such as in chemical sensors, solar cells, and photocatalysts [6]. Fullerene nanotubes (FNTs) as cylindrical nanostructure have advantages such as high tensile strength and electrical conductivity, facilitating their use in nanocomposites and nanoelectronics [7].

Nowadays, fullerenes have found significant potential in the realm of biomedical applications, including antioxidants, photodynamic therapy, biosensors, imaging, and drug delivery [8]. Self-assembled FNWs were found to promote the orientation and differentiation of neural stem cell [9]. FNTs can enhance electrical conductivity in tissue engineering scaffolds to facilitate the repair of ischemic heart tissue [10]. However, the hydrophobic nature of fullerene has caused significant limitation when utilized in various biomedical applications [11]. The hydrophilic modification strategy for hydrophobic fullerene presents an opportunity to study the effect of fullerene nanoarchitectonics on cells cultured in two-dimensional (2D) or three-dimensional (3D) environments [12]. Encapsulation of fullerenes in amphiphilic carriers like micelles and liposomes can simultaneously enhance water solubility and maintain membrane-like properties [13]. Pluronic F127 as an amphiphilic carrier containing micellar cavities was used to encapsulate fullerene to improve water solubility and cellular uptake with minimal cytotoxicity for cancer therapy [14]. FNTs-encapsulated Pluronic F123 can stimulate the endocytic activity of neural stem cells (NSCs) through morphological change in 2D culture and promote the differentiation of NSCs when used in combination with hydrogels in 3D culture [15].

Hydrogels have been explored as versatile platforms in a wide range of biomedical applications through diverse structural, physicochemical, and biological properties [16]. In the therapeutics associated with angiogenesis, hydrogels provide microenvironments similar to the natural extracellular matrix (ECM) to enable the formation of microvessels with endothelial cells (ECs) in vitro [17]. Especially, biodegradable hydrogels with soft stiffness facilitate angiogenic sprouting and microvessel formation because stronger cell-cell contact than cell-ECM interactions [18]. Self-healing hydrogels are suitable materials for angiogenic therapeutics with tunable mechanical properties through adjustment of the dynamic network [19]. Self-healing hydrogels based on chitosan are developed through Schiff base linkages to promote in vivo chronic wound healing with angiogenesis [20]. Moreover, chitosan-based self-healing hydrogels have in vivo angiogenic capacities to rescue the blood circulation in ischemic hindlimbs of mice with good biocompatibility and biodegradability [21]. However, the angiogenic microvessels in these self-healing hydrogels grew randomly without specific perfusion and orientation. Also, the mechanisms that determine the spatial organization of angiogenic growth in self-healing hydrogels have not been studied extensively.

Mechanical forces may impact the cellular motility, metabolism, and proliferation of ECs and contribute to the advancement of angiogenesis [22]. The reaction of ECs to mechanical cues is modulated by the composition and structure of the ECM, which is an initial mediator of cellular mechanotransduction [23]. Self-healing hydrogels incorporated with nanomaterials may change physicochemical characteristics and provide mechanical strain for traction and orientation for angiogenic microvessels [24]. In this study, we developed chitosan-based self-healing hydrogels containing hydrophilic fullerenes as in vitro 3D platforms of angiogenesis. Meanwhile, we aimed to investigate different mechanical boundary conditions, which was affected by FNWs and FNTs, in the regulation of angiogenic growth and orientation with ECs. This study may offer a possible rationale for designing the naturally occurring aligned vascular networks in tissues and lay the foundation for enhanced control over vascularization in native tissues and tissue-engineered constructs.

## 2. Materials and methods

### 2.1. Synthesis of hydrophobic fullerene self-assemblies of FNTs and FNWs

The synthesis of fullerene self-assemblies involved the utilization of the modified liquid-liquid interfacial precipitation (LLIP) method. Hydrophobic FNTs and FNWs were fabricated using distinct crystallization techniques at the liquid-liquid interface by mixing various concentrations of 99.5% pure fullerene powder (MTR Ltd, U.S.A.) dissolved in different aromatic solvents with non-solvent alcohols. In the typical preparation process of FNTs, a saturated solution of fullerene in m-xylene (Wako, Japan) was prepared by treating an excess of pristine fullerene powder (100 mg) with 100 mL of m-xylene (1 mg/mL). Undissolved fullerene was removed by filtration. Subsequently, 3 mL of 2-propanol (IPA, Wako, Japan) was placed in a clean, dry glass vial (13.5 mL), and the saturated solution of fullerene in m-xylene (1 mL) was rapidly added. The resulting mixture was incubated at 25°C for 1 h to facilitate crystal growth, leading to a blackish appearance due to FNT formation. The resulting FNTs were initially purified by centrifugation at 3000 rpm for 3 min, with the precipitate containing FNTs and the supernatant containing unreacted fullerene being separated. The FNTs were then washed once with IPA and subjected to centrifugation under the same conditions. This purification process was repeated three times, and the resulting precipitate was vacuum-dried at 80°C for 24 h. The preparation method for FNWs closely followed a similar procedure to that of FNTs. In a typical preparation process of FNWs, a saturated solution of fullerene in toluene (Wako, Japan) was prepared by treating an excess of pristine fullerene powder (320 mg) with 100 mL of toluene (3.2 mg/mL). Undissolved fullerene was filtered out. Afterwards, a portion of the saturated solution of fullerene in toluene (1 mL) was placed in a clean, dry glass vial (13.5 mL) and cooled down to 4°C in a temperature-controlled incubator for 2 h. Then, 5 mL of IPA, which had also been precooled at 4°C for 2 h, was slowly layered on top of the saturated fullerene solution in toluene. The resulting mixture was incubated at 4°C for 30 min. Gentle sonication was applied for 1 min in a bath sonicator and the mixture was again incubated at 4°C for 24 h to grow the crystals. The mixture solution turned brownish due to the formation of FNWs. The resulting FNWs were first purified by centrifugation at 3000 rpm for 3 min, whereby FNWs precipitated and the supernatant containing unreacted fullerene was discarded. The FNWs were then washed once with IPA and subjected to centrifugation under the same conditions. After repeating the above step three times, the precipitate was vacuum-dried at 80°C for 24 h.

### 2.2. Preparation of hydrophilic fullerene self-assemblies

To impart surface hydrophilicity to the hydrophobic fullerene self-assemblies, Pluronic (*p*-123, polyethylene glycol-block-polypropylene glycol-block-polyethylene glycol, number average molecular weight (Mn)∼ 5800, Sigma-Aldrich, U.S.A.) was used to treat FNTs or FNWs for surface modification. Briefly, the FNTs or FNWs powder was directly added into Pluronic aqueous solution (0.5 wt%) and left to resuspend overnight to obtain FNTs-Pluronic (FNT-P) or FNWs-Pluronic (FNW-P). After centrifugation and removal of the supernatant, the precipitate was washed with ultrapure water. This washing step was repeated twice times, and the precipitate was vacuum-dried at 80°C for 24 h.

### 2.3. Characterization of hydrophobic and hydrophilic fullerene self-assemblies

The morphologies of hydrophobic (FNT and FNW) and hydrophilic (FNT-P and FNW-P) fullerene self-assemblies were characterized using scanning electron microscopy (SEM, S-4800, Hitachi. Japan). The equipment operated at an accelerating voltage of 10 kV. Before measurement, the fullerene powders were suspended in IPA or distilled water (1 mg/mL) to form a mixture and then respectively deposited onto a passivated silicon wafer and dried. The SEM samples were subsequently coated with a conductive layer of approximately 2 nm platinum metal using a Hitachi S-2030 ion coater for image observation. The size determination of FNT-P and FNW-P was conducted from the SEM images using ImageJ software (National Institutes of Health, Bethesda, MD).

Surface functional groups were characterized using attenuated total reflection-Fourier transform infrared (ATR-FTIR, Nexus 670, Japan) spectroscopy. Additionally, the surface charge of the fullerene self-assemblies was assessed through zeta potential measurements employing a Zetasizer (ZS ZEN 3600, Malvern, UK).

The sessile drop technique was employed to assess the water contact angle of hydrophobic and hydrophilic fullerene self-assemblies using a contact angle meter (FTA-1000B, First Ten Angstrom Company, U.S.A.). For evaluation, a droplet volume of 3 μL was used, and a waiting period of 90 seconds was observed before measuring the contact angle.

### 2.3. Preparation of chitosan self-healing hydrogels and the composite hydrogels with self-assembled fullerenes

Chitosan self-healing hydrogels (CD) containing dynamic Schiff-base linkages were prepared by blending 1–2.5 wt% O-carboxymethyl chitosan (Biosynthcarbosynth, ~100 kDa, deacetylation degree 78.2%, UK) with a 1 wt% dynamic crosslinker (polyethylene oxide capped with benzaldehyde, DPEO, 4 kDa) at room temperature. The DPEO was synthesized through a Steglich esterification reaction involving PEG (Sigma-Aldrich, 4000 kDa) and 4-formylbenzoic acid (Sigma-Aldrich) to generate telechelic aldehyde groups, which is followed by a procedure in previous literature [25].

To prepare the composite hydrogels, FNT-P and FNW-P with different concentration (25, 50, and 100 µg/mL) were incorporated into the 2 wt% CD hydrogels. The macroscopic self-healing ability was initially assessed by merging two pieces of hydrogel, and one piece of the hydrogel was stained with methylene blue, while the other piece left unstained for observation. The injectability was evaluated by pushing the hydrogel through a syringe with tiny needles (33-gauge, ~108 µm internal diameter).

### 2.4. Rheological properties of hydrogels

The viscoelastic characteristics of the hydrogels were examined using a rheometer (RS-5, TA Instruments, U.S.A.) equipped with a cone-plate geometry. The measurements were conducted immediately after placing the hydrogel samples (700 µL) on the platform, while maintaining the samples at 37°C. The storage shear modulus (G’) and loss shear modulus (G’’) were measured over time (0–3000 seconds) at a frequency of 1 Hz and a 1% oscillatory strain (time sweep). The strain sensitivity, which represents the strain level at which damage occurs in each hydrogel, was assessed at a frequency of 1 Hz using an incremental stepwise approach, ranging from 1% to 1000% oscillatory shear strain (referred to as a strain sweep test). To evaluate the self-healing properties, damage-healing cycles were conducted following the results of the strain sweep. The cyclic damage-healing steps were carried out at a dynamic constant frequency of 1 Hz within the lower strain (1%) and the higher strain (where damage had occurred) based on the strain sweep test of each hydrogel. Subsequently, a static shear experiment was performed to measure the steady shear viscosities while increasing the shear rates from 1 to 100 s^−1^.

### 2.5. Culture of rat ECs

Rat ECs were isolated from the carotid artery of adult Sprague-Dawley rats. The cells were cultured in EGM-2 medium (Lonza, BulletKitTM, U.S.A.) with 10% fetal bovine serum at 37°C in a humidified environment with 5% CO_2_. The culture medium was refreshed every 48 hours, and cell passaging was conducted when the culture reached 80%–90% confluence. Rat ECs from the fourth passage were employed in the following experiments.

### 2.6. In vitro *cell viability and proliferation of ECs in the presence of self-assembled fullerenes for 2D culture and the composite hydrogels for 3D culture*

The cell viability and proliferation of ECs in a 2D and 3D environment were assessed by Cell Counting Kit-8 (CCK-8, Sigma, U.S.A). For the 2D environment, ECs (10^4^/well) were cultured with the cell culture medium containing FNT-P or FNW-P (25, 50, and 100 µg/mL) in the wells of a 24-well culture plate for 7 days. For the 3D environment, ECs (10^6^ cells/mL) were cultured in the CD hydrogels incorporated with FNT-P or FNW-P (25, 50, and 100 µg/mL) for 7 days. For the cell viability test, ECs were seeded on the wells for 2D culture or mixed with the hydrogels for 3D culture and incubated for 24 h. Afterwards, the culture medium was changed to a medium with 50 μL of the CCK-8 solution and 950 μL of the culture medium, which followed the instructions of the manufacturer. After reaction for 2 h, the absorbance optical density (O.D.) of the medium with CCK-8 solution was measured at 450 nm using a microplate reader (SpectraMax iD3, Molecular Devices, U.S.A.). The cell viability was determined as (O.D. sample/O.D. control) × 100%, which was the percentage relative to the control group. The control groups were those cultured in the culture medium without FNT-P or FNW-P for 2D culture and those in CD hydrogels without FNT-P or FNW-P for 3D culture.

For cell proliferation test, ECs were seeded on the wells for 2D culture or mixed with the hydrogels for 3D culture and incubated for 4 h at the first day (day 0). After ECs attached on the well or the hydrogels, the culture medium was changed to the medium with CCK-8 solution. After reacted for 2 h, the first absorbance of each sample (O.D. initial) of the medium with CCK-8 solution was measured at 450 nm. Then, the medium with CCK-8 solution were removed and replaced with the fresh culture medium for the subsequent culture. After 3 and 7 days of culture, the absorbance O.D. of each sample (O.D. final) was detected as mentioned above. The proliferation rate of ECs was determined as (O.D. final/O.D. initial) × 100%, which was the percentage relative to the initial value of each sample.

The activity of reactive oxygen species (ROS) in ECs following interaction with hydrogels containing FNT-P or FNW-P was assessed through a cell vitality assay using an advanced image cytometer (NucleoCounter NC-3000, ChemoMetec, Denmark). ECs were initially cultured in 24-well plates, with 1 × 10^5^ cells per well, and left to grow overnight. Subsequently, they were incorporated into hydrogels with different concentrations of FNT-P or FNW-P (25, 50, and 100 µg/mL) for 1 h. Afterwards, the ECs were dissociated and collected through accumax. The resulting cell pellet was re-suspended with 50 μL of culture medium. Next, a 19 μL aliquot of the cell suspension was stained with 1 μL of Solution 5 (ChemoMetec, Denmark), following the guidelines of the manufacturer. Solution 5 consisted of three types of fluorescent stains: propidium iodide to stain dead cells, acridine orange as a fluorescent counterstain, and VitaBright-48 (VB-48) to indicate the levels of thiols such as reduced glutathione in the cells. Finally, 10 μL of the stained cells were loaded into an 8-chamber NC-Slide A8™ and analyzed using the image cytometer system.

### 2.7. Immunofluorescence staining

For observation of angiogenic growth and orientation with ECs in the hydrogels containing FNT-P or FNW-P, the EC-incorporated hydrogels were stained for platelet endothelial cell adhesion molecule (CD31) by immunofluorescence staining after culture for 7 d. ECs (10^6^ cells/mL) were incorporated in hydrogels containing FNT-P or FNW-P (50 µg/mL) and cultured at 37°C for 7 days. After 7 days, the ECs in the hydrogels were fixed in 4% paraformaldehyde for 20 min. Afterwards, the samples were blocked with 0.5% BSA at 25°C for 1 h, followed by incubation at 4°C for 24 h with CD31 (Invitrogen, U.S.A.) antibodies. Then, the ECs were counterstained with Hoechst 33,258 (the fluorescent nuclear stain, Sigma-Aldrich) and visualized using a fluorescence microscope (Leica, DM IRB, Germany).

### 2.8. Gene expression for cells encapsulated in hydrogels

The expression of vascular-related genes for ECs cultured in CD hydrogels and composite hydrogels of CD with FNT-P or FNW-P (50 µg/mL) for 7 days was analyzed using the KAPA SYBR Green qPCR kit (Kapa Biosystem, Inc., UK) through the real-time reverse transcriptase-polymerase chain reaction (RT-PCR). The data were obtained using a Step One Plus Real-Time PCR instrument (Applied Biosystems, U.S.A.). Expression levels were normalized to 3-phosphoglycerate dehydrogenase (GAPDH, the housekeeping gene). The vascular markers in the study included endothelial nitric oxide synthase (eNOS), vascular endothelial growth factor receptor (VEGFR), C-X-C motif chemokine receptor 4 (CXCR4), stromal cell-derived factor-1 (SDF-1), CD31, and Ras homolog family member A (RhoA). The primer sequences for each gene are detailed in Table 1.

**Table 1.** The primer sequences used for RT-PCR analyses of ECs.

Gene	Primer sequences	Annealing temperature (°C)
Rat GAPDH	F: 5’-CTCAGTTGCTGAGGAGTCCC-3’ R: 5’-GGTATTCGAGAGAAGGGAGG-3’	60
Rat eNOS	F: 5’-GGTTACCATGAGTTCAGGGCCAGC-3’ R: 5’-GCATGTGTGTGCATATGTGTAAGA-3’	60
Rat VEGFR	F: 5’-TGACACGGAAACTGAAGACC-3’ R: 5’-TTGGAGTTTCAGAGGCAGGT-3’	60
Rat SDF-1	F: 5’-TCCGCTTCTCACCTCTGTAG-3’ R: 5’-GCTGGCTCCATTCTACAGGA-3’	60
Rat CXCR4	F: 5’-GTGTGTGTGTGTGTGAGGTT-3’ R: 5’-ACAGCTGAGGGTCACTTCTA-3’	60
Rat CD31	F: 5’-CTTCACCATCCAGAAGGAAGAGAC-3’ R: 5’-CACTGGTATTCCATGTCTCTGGTG-3’	60
Rat RhoA	F: 5’-GCCAAAATGAAGCAGGAGCC-3’ R: 5’-TACCCAAAAGCGCCAATCCT-3’	60

### 2.9. Statistical analysis

The experimental data were presented as the mean values along with their corresponding standard deviations. The reproducibility of each experiment was independently validated through three repetitions. To assess the statistical distinctions between the experimental groups, a one-way ANOVA was conducted. Statistically significant differences were denoted by asterisks, with *p < 0.05, **p < 0.01, and ***p < 0.001 indicating various levels of significance.

## 3. Results and discussion

### 3.1. Preparation of hydrophobic and hydrophilic self-assembled fullerenes and their morphologies

**Figure 1.**
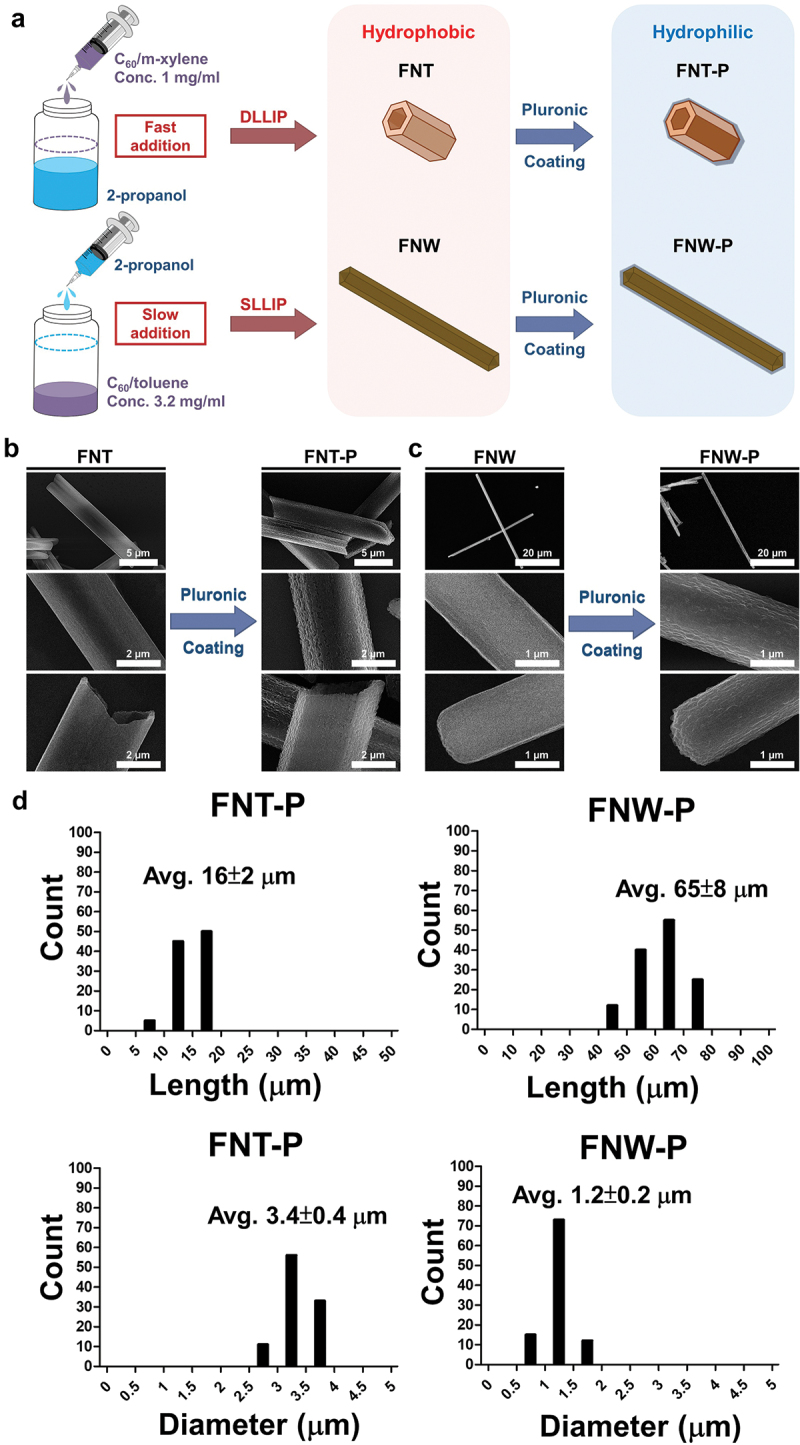
Synthesis of hydrophobic fullerene self-assemblies and the surface modification by pluronic to yield hydrophilic fullerene self-assemblies. (a) The schematic details the preparation of FNT, FNW, FNT-P, and FNW-P. (b,c) Scanning electron microscope (SEM) images were taken before (FNT and FNW) and after the surface modification by pluronic (FNT-P and FNW-P). (d) The size distribution of hydrophobic self-assembled fullerenes (FNT and FNW) and hydrophilic self-assembled fullerenes (FNT-P and FNW-P) after the surface modification by pluronic.

The nanoarchitectured structures of fullerene were produced through self-assembly by the LLIP method [4]. These nanostructures result from the supramolecular assembly of fullerene molecules due to π–π stacking interactions. Typically, the LLIP technique involves an inter-diffusion procedure between a solution of fullerene in a good solvent and a poor solvent. This process occurs by layering the fullerene solution in the good solvent onto the poor solvent, leading to the precipitation of one-dimensional (1D) fullerene crystals with specific shapes [6]. In the dynamic LLIP methods, inter-diffusion is assisted by manual mixing or ultrasonication [5]. Respectively, the inter-diffusion for the static LLIP method occurs spontaneously without manual mixing or ultrasonication [26]. The preparation of hydrophobic and hydrophilic self-assembled fullerenes used in this study is illustrated in Figure 1(a). The FNTs with a smaller aspect ratio were prepared using the dynamic LLIP method with m-xylene as good solvent and IPA as poor solvent. Each extreme of the tube showed a cavity deep into one-third of the overall length due to the difference in crystal-growth rates between the edge and center of the surface of fullerene seed crystals, which formed soon after nucleation [27]. Meanwhile, the FNWs were prepared using the static LLIP method with toluene as good solvent and IPA as poor solvent. As toluene diffuses into IPA, the solution became supersaturated, which caused fullerene to be limited at the IPA-toluene interface. FNWs formed as a result of low solubility of fullerene in IPA. Meanwhile, the kinetically controlled crystal growth led to FNWs growing parallel to the [110] direction [28]. SEM images for the self-assembled fullerenes and those after hydrophilic modification are demonstrated in Figure 1(b,c). After surface coating with the hydrophilic Pluronic layer, the modified fullerenes (i.e. FNT-P and FNW-P) became hydrophilic and were uniformly dispersed in the aqueous solution. The histograms depicting the size distribution of FNT-P and FNW-P are shown in Figure 1(d). The average length and diameter of FNT-P were 16 ± 2 µm and 3.4 ± 0.4 µm (i.e. an aspect ratio of 4.6), respectively. The average length and diameter of FNW-P were 65 ± 8 µm and 1.2 ± 0.2 µm (i.e. an aspect ratio of 53.9), respectively. Moreover, no size difference was observed between self-assembled fullerenes before and after hydrophilic modification.

### 3.2. Characterization of hydrophobic and hydrophilic self-assembled fullerenes

**Figure 2.**
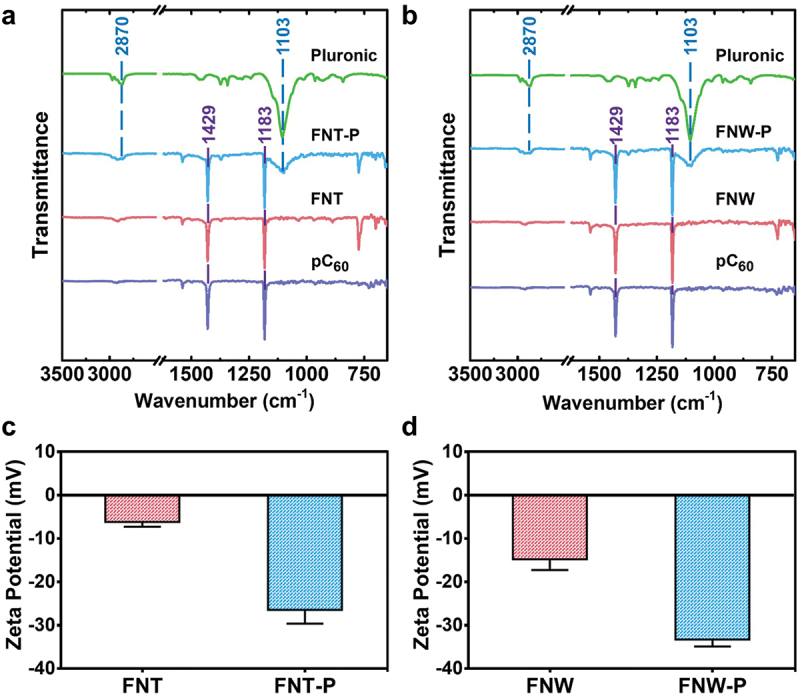
Characterization of the hydrophobic and hydrophilic fullerene self-assemblies. (a) Fourier-transform infrared spectroscopy (FT-IR) profiles, measured using the attenuated total reflection (ATR) technique, for the fullerene self-assemblies before and after pluronic coating. (b) Comprehensive zeta potential analyses highlight the surface charge variations among FNT, FNW, FNT-P, and FNW-P.

In Figure 2(a), the ATR-FTIR spectra of pristine fullerene, FNTs, FNWs, FNT-P, and FNW-P revealed two prominent peaks at 1183 and 1429 cm^−1^, which confirmed the predominant presence of fullerene molecules in these samples [29]. Pluronic exhibited peak positions at 1103 and 2870 cm^−1^, and these same peaks were evident in FNT-P and FNW-P, proving the successful coating of Pluronic on FNT-P and FNW-P [30]. The zeta potentials of FNTs, FNWs, FNT-P, and FNW-P were measured, and results are shown in Figure 2(b). The zeta potentials of FNTs and FNWs were −6.5 ± 1.2 mV and −15 ± 2 mV, respectively. The zeta potential difference between FNTs and FNWs refers to the variation in electrical charge at their respective surfaces [31]. FNWs have a greater specific surface area than FNTs, which may cause the FNWs to exhibit more negative zeta potential. After the hydrophilic modification with Pluronic, the zeta potentials of FNT-P and FNW-P decreased to −15 ± 2 mV and −34 ± 2 mV, respectively, which indicated successful coating of Pluronic on FNT-P and FNW-P. Moreover, the FNT-P and FNW-P may have increased aqueous stability due to the presence of Pluronic on the surface with more negative zeta potential [32]. The variation in hydrophilic and hydrophobic self-assembled fullerenes was verified by applying an equal quantity of self-assembled fullerenes on a slide and then measuring the water contact angle (Figure S1(a)). The contact angle was reduced from 113.5 degrees for FNT to 18.7 degrees for FNT-P. Meanwhile, the contact angle was reduced from 132.2 degrees for FNW to 22.0 degrees for FNW-P. In addition, the stability of the coating layer on the self-assembled fullerenes in cell culture medium is presented in Figure S1(b). These results suggest the successful Pluronic coating on the self-assembled fullerenes, transforming the originally hydrophobic fullerenes into a more hydrophilic state. The stability of the coating layer was confirmed by the absence of significant change in the surface water contact angle after immersion in cell culture medium for 7 days.

### 3.3. Preparation of chitosan self-healing hydrogels and the composite hydrogels with self-assembled fullerenes

**Figure 3.**
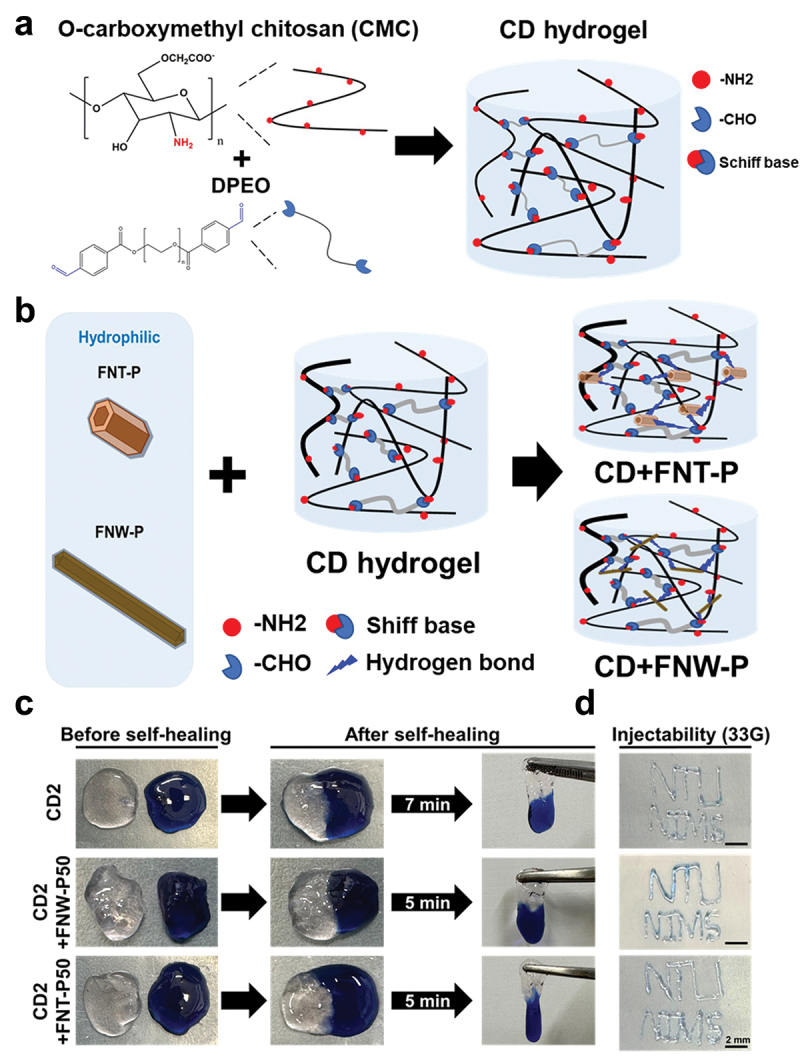
Preparation of chitosan self-healing hydrogels and the composite hydrogels with self-assembled fullerenes. (a) Schematic illustration of polyethylene oxide capped with benzaldehyde (DPEO) as the crosslinker that reacts with the amine group on O-carboxymethyl chitosan (CMC) to form self-healing hydrogels (CD hydrogels; including CD2.5, CD2, CD1.5, and CD1). (b) Schematic illustration of the composite hydrogels made from a CD hydrogel and self-assembled fullerenes of different nanoarchitectural structures (i.e. FNT-P or FNW-P). (c) Images showing the self-healing ability of the CD hydrogel (CD2) and the composite hydrogel with self-assembled fullerenes. (d) Injection of the CD2 self-healing hydrogel and the composite hydrogels to pass through a 33-gauge syringe needle. Scale bar: 2 mm.

Several chitosan self-healing hydrogels with dynamic covalent bonding (CD hydrogels) were prepared for this study. The chemical composition is illustrated in Figure 3(a). Meanwhile, a schematic for preparing composite hydrogels from CD and self-assembled fullerenes is depicted in Figure 3(b). The amino group on CMC underwent a reaction with benzaldehydes located at both ends of DPEO, resulting in the formation of a dynamically crosslinked network for the self-healing hydrogel. As shown in Figure 3(b), the hydrophilic layer of Pluronic was at the surface of FNT-P and FNW-P, which indicated that the hydroxyl groups of FNT-P and FNW-P may form hydrogen bond with CD hydrogel to generate the composite hydrogels [33]. The successful hydrophilic coating allows the self-assembled fullerenes to be dispersed more homogeneously and stably in water, cell culture media, and hydrogels, which facilitates the analysis of mechanical properties and in vitro cell experiments of the composite materials (Figure S2). Pluronic-coated materials, also known as poloxamers, enhance the hydrophilicity through their triblock copolymer structure that features a central hydrophobic poly(propylene oxide) block and two hydrophilic PEO blocks [34]. This structure enables the formation of hydrogen bonds between the water-friendly PEO chains and water molecules. Hydrogen bonds enhance the hydrophilicity and stability of materials in aqueous environments, which is ideal for biomedical applications [35].

The macroscopic self-healing capability and injectability of the CD hydrogel and the composite hydrogels with self-assembled fullerenes are shown in Figure 3(c,d). The CD hydrogel and composite hydrogels were each cut into two pieces, and two segments were put together, ensuring that the cut surfaces remained in contact. Within 10 minutes, the two segments of the CD hydrogel (or the composite hydrogel) mended to form a unified hydrogel, and the cracks between the two segments vanished. The healed CD hydrogel and composite hydrogels were pulled to fracture, while the fractures were not at the original cracks, which indicated that both the CD hydrogel and the composite hydrogels had self-healing capability. The injectability test revealed that FNW-P and FNT-P at the concentration of 50 µg/mL did not adversely affect the injectability of the composite hydrogels through a 33-gauge tiny syringe needle (~108 µm internal diameter).

### 3.4. Rheology of CD hydrogels

**Figure 4.**
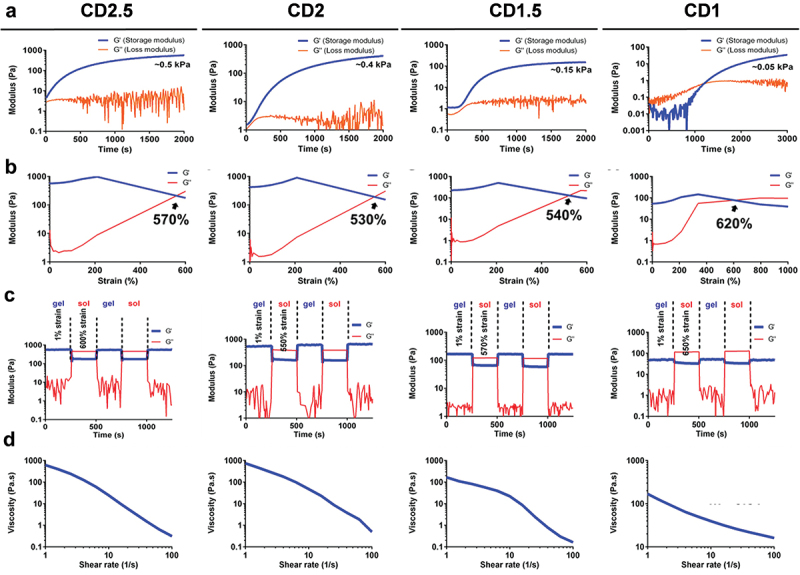
Rheological properties of CD hydrogels with various composition at 37°C. (a) Time-sweep experiments showing the storage moduli and loss moduli (G’ and G”) of the CD hydrogels against the gelling time at 1 hz frequency and 1% dynamic strain. (b) The strain-dependent G’ and G” of the CD hydrogels at 1 hz frequency. (c) Self-healing experiments showing the G’ and G” values of the equilibrium hydrogels at 1 hz frequency and alternate continuous damage-healing cycles of 1% and damaged strain (%) of each CD hydrogel. (d) The steady shear-thinning properties of the CD hydrogels determined by measuring the viscosity versus shear rate from 1 to 100 s^−1^.

The mechanical properties of CD hydrogels with various compositions were investigated by rheological measurements, as shown in Figure 4. The component ratios of CD hydrogels are listed in Table 2. The time-dependent and strain-dependent viscoelasticity of the hydrogels are exhibited in Figure 4(a,b). The equilibrium modulus and damaged strain of the hydrogels varied as the weight percent of CMC decreased, indicating that the hydrogel system possessed tunable mechanical properties [36]. The mechanical properties of a hydrogel can affect many cellular behaviors, including cell migration, spreading, and differentiation [37]. The G’ value of CD2.5 approached ~0.5 kPa after gelation, which was the highest among all groups. The G’ of the hydrogels decreased as the weight percent of CMC decreased, and the values were ~ 0.4 kPa, ~0.15 kPa, and ~ 0.05 kPa for CD2, CD1.5, and CD1, respectively. Meanwhile, the oscillation observed in G’’ may be connected to the breaking and reforming of dynamic Schiff base linkages within the gel network [38]. The damaged strain took place at approximately 570%, 530%, 540%, and 620% for the CD2.5, CD2, CD1.5, and CD1 hydrogels, respectively. The damage-healing cycles demonstrated that the self-healing efficiency reached nearly 100% of all hydrogels, as displayed in Figure 4(c). The steady shear viscosity of the hydrogels under different shear rates is displayed in Figure 4(d). All hydrogels revealed good shear-thinning behavior, which indicates the suitability of the hydrogels for injection applications [39]. These functionalities suggest that the hydrogel system have potential for 3D-printing and minimal invasive procedures [40].

**Table 2.** Compositions of various CD hydrogels. CMC: O-carboxymethyl chitosan, DPEO: polyethylene oxide capped with benzaldehyde.

CD hydrogel	CMC (wt%)	DPEO (wt%)
CD2.5	2.5	1.0
CD2	2.0	1.0
CD1.5	1.5	1.0
CD1	1.0	1.0

### 3.5. Rheology of the composite hydrogels containing self-assembled fullerenes

**Figure 5.**
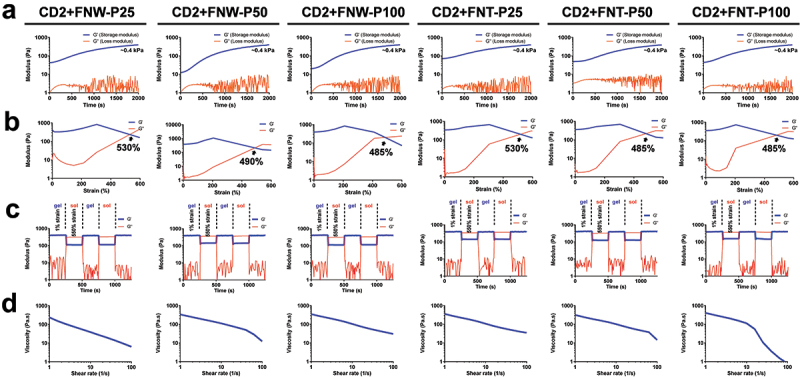
Rheological properties of the composite CD hydrogels with different concentrations (25, 50, and 100 µg/mL) of self-assembled fullerenes (FNT-P or FNW-P). (a) Time-sweep experiments showing the storage moduli and loss moduli (G’ and G”) of the composite CD hydrogels against the gelling time at 1 hz frequency and 1% dynamic strain. (b) The strain-dependent G’ and G” of the composite CD hydrogels at 1 hz frequency. (c) Self-healing experiments showing the G’ and G” values of the equilibrium hydrogels at 1 hz frequency and alternate continuous damage-healing cycles of 1% and damaged strain (%) of each composite CD hydrogel. (d) The steady shear-thinning properties of the composite CD hydrogels determined by measuring the viscosity versus shear rate from 1 to 100 s^−1^.

The enhancement of material properties in manufacturing may rely on the creation of composites that leverage the additive or synergistic characteristics of their component materials [7]. In the current study, the mechanical properties of the composite hydrogels with self-assembled fullerenes are shown in Figure 5. The component ratios of the composite hydrogels are listed in Table 3. In Figure 5(a), the equilibrium modulus of all hydrogels was ~0.4 kPa, which was similar to that of CD2 without fullerenes. This finding indicated that incorporating a small amount of self-assembled fullerenes may not affect the mechanical properties of the hydrogels. However, the damaged strain of the hydrogels occurred at lower strains when over 50 µg/mL of self-assembled fullerenes was added, as shown in Figure 5(b). This finding suggests that fullerenes may cause structural change in the composite hydrogels, thereby affecting the susceptibility of the hydrogels to damage of lower strains and imparting some enhancement in self-healing properties. Moreover, the enhanced self-healing properties of the hydrogels may facilitate diffusion and mass transfer in the surrounding environment, which increased nutrients and oxygen exchange for cell growth and promoted cell attachment and spreading [41]. The self-healing efficiency of all composite hydrogels containing self-assembled fullerenes approached nearly 100% during the damage-healing cycles, as demonstrated in Figure 5(c). Meanwhile, all hydrogels remained good shear-thinning behavior, as presented in Figure 5(d). The results suggest that the addition of hydrophilic fullerenes may introduce more hydrogen bonding into the system without compromising self-healing properties and injectability, which may make the entire system more biomimetic [42].

**Table 3.** Component ratios of the composite hydrogels with self-assembled fullerenes in various contents.

Hydrogel	CMC (wt%)	DPEO (wt%)	Contents of self-assembled fullerenes (µg/mL)
CD2+FNT-P25	2.0	1.0	25
CD2+FNT-P50	2.0	1.0	50
CD2+FNT-P100	2.0	1.0	100
CD2+FNW-P25	2.0	1.0	25
CD2+FNW-P25	2.0	1.0	50
CD2+FNW-P25	2.0	1.0	100

### 3.6. In vitro *cell viability, proliferation, and morphology with self-assembled fullerenes*

**Figure 6.**
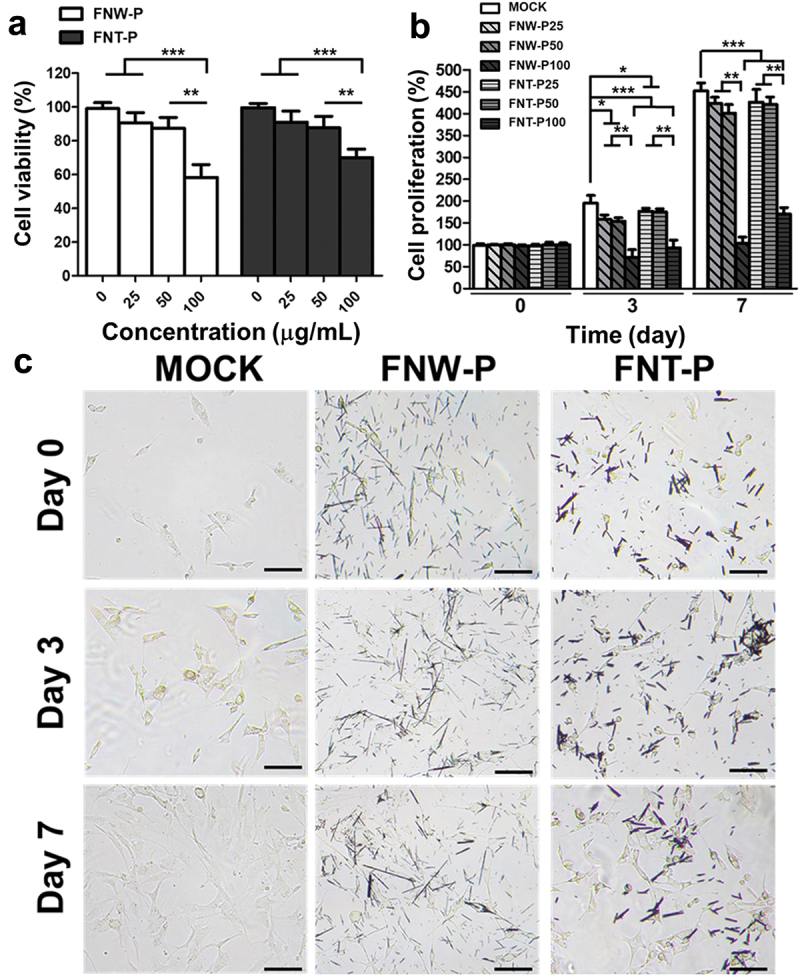
*In vitro* survival, proliferation, and morphology of ECs in the presence of self-assembled fullerenes (FNT-P or FNW-P) for the 2D culture. (a) Cell viability of ECs after culture with different concentrations of FNT-P or FNW-P for 24 h, measured by a CCK-8 assay. The cell viability was determined as (O.D. sample/O.D. control) × 100%, which was the percentage relative to the control group. The control groups were ECs cultured in the culture medium without FNT-P or FNW-P for 2D culture. (b) Long-term proliferation of ECs with different concentrations of FNT-P or FNW-P, measured by CCK-8 assay after a culture period of 0, 3, and 7 days. The proliferation was normalized to the value at day 0 and performed as the percentage of the cell proliferation (%). (c) Observation of the morphology and growth of ECs after culture with different concentrations of FNT-P or FNW-P in the 2D environment after a culture period of 0, 3, and 7 days. Scale bar: 100 µm. Asterisks indicate statistically significant differences, *p < 0.05, **p < 0.01, and ***p < 0.001.

The viability and proliferation of ECs after treatment with self-assembled fullerenes (FNW-P or FNT-P) of different concentrations (25, 50, and 100 µg/mL) in the 2D environment were evaluated by the CCK-8 assay, as displayed in Figure 6(a,b). After 24 h, the cell viabilities of ECs were respectively ~ 90% and ~88% for FNW-P25 and FNW-P50 and respectively ~90% and ~88% for FNT-P25 and FNT-P50, which did not show significant difference in comparison to the control group (i.e. culture medium without fullerenes). However, the apoptosis of ECs could occur at higher concentrations of fullerenes, and the cell viabilities were ~58% for FNW-P100 and ~69% for FNT-P100, which were significantly lower than those of the control group and the groups with FNW-P25, FNW-P50, FNT-P25, and FNW-P50. For long-term culture after 3 days, the proliferation rate of ECs in the control group (~187%) was significantly higher than those of the groups with FNW-P25 (~152%), FNW-P50 (~150%), FNW-P100 (~80%), FNT-P25 (~170%), FNW-P50 (170%), and FNW-P100 (~87%). After culture for 7 days, the proliferation rates of ECs in the groups with FNW-P25, FNW-P50, FNT-P25, and FNT-P50 were ~422%, ~400%, ~424%, and ~420%, respectively, which showed no significant difference from that of the control group (~440%). However, the proliferation rates of ECs in the groups with FNW-P100 and FNT-P100 were ~105% and ~ 168%, respectively, which were significantly lower than those of the control group and the groups with FNW-P25, FNW-P50, FNT-P25, and FNT-P50. Taken together, these results indicated that ECs remained viable and proliferating in the presence of low concentration fullerenes.

Moreover, different nanoarchitectured structures of fullerenes showed the potential to affect the morphology and the growth of ECs upon interaction with ECs, as shown in Figure 6(c). The ECs revealed flattened and elongated morphology and grew in alignment with the arrangement of the FNW-P. Meanwhile, the ECs displayed relatively rounded and entwined morphology, and tended to form clusters and grow in association with the FNT-P. This observation suggests that the presence of fullerenes may modulate the morphological changes in ECs and further affect their proliferation through nanotopography-mediated cellular forces that potentially trigger signal transduction pathways [43]. This mechanism is presumably initiated by activating cell surface integrin receptors and focal adhesions, which relay cues from the ECM to the cytoskeleton [44].

### 3.7. In vitro *cell viability and proliferation in the composite hydrogel*

**Figure 7.**
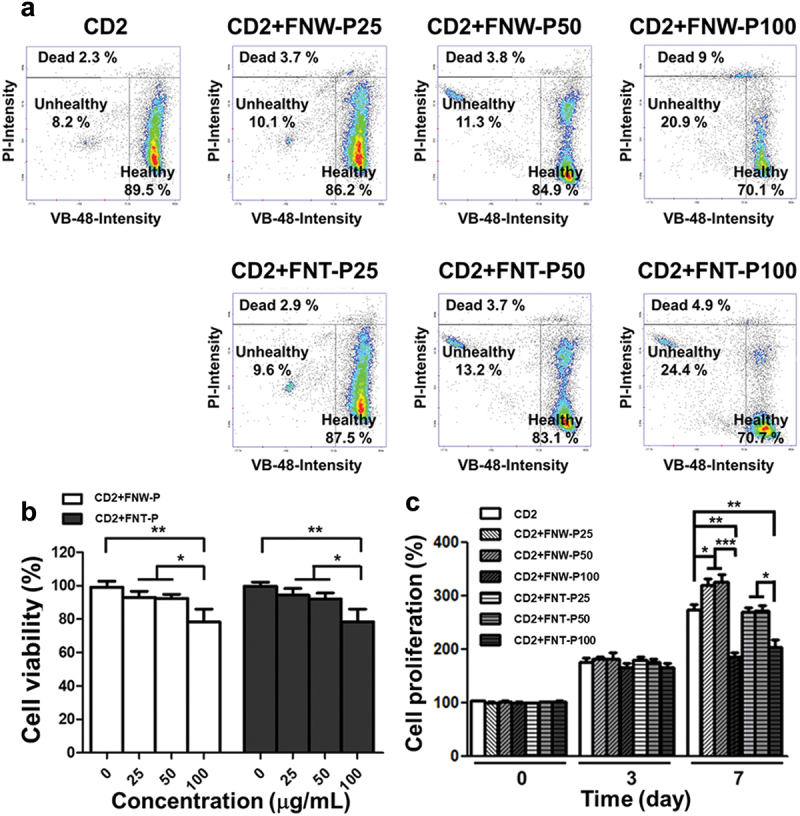
*In vitro* survival and proliferation of ECs in the composite CD hydrogels for the 3D culture. (a) The vitality of ECs was analyzed by the vitabright-48 staining assay after exposure to the composite CD hydrogels with FNT-P or FNW-P of different concentrations for 1 h. (b) Cell viability of ECs after culture in the composite CD hydrogels with FNT-P or FNW-P of different concentrations for 24 h, measured by a CCK-8 assay. The cell viability was determined as (O.D. sample/O.D. control) × 100%, which was the percentage relative to the control group. The control groups were ECs in the composite CD hydrogels without FNT-P or FNW-P for 3D culture. (c) Long-term proliferation of ECs in the composite CD hydrogels with different concentrations of FNT-P or FNW-P, measured by CCK-8 assay after a culture period of 0, 3, and 7 days. The proliferation was normalized to the value at day 0 and performed as the percentage of the cell proliferation (%). Asterisks indicate statistically significant differences, *p < 0.05, **p < 0.01, and ***p < 0.001.

The VB-48 staining assay and CCK-8 assay were employed to assess the survival of ECs in CD2 hydrogels containing FNW-P or FNT-P, as depicted in Figure 7(a,b). Cytometric analyses showed that the glutathione (GSH) levels (indicative of cell health) in CD2 hydrogels containing FNW-P25, FNW-P50, FNT-P25, and FNT-P50 were nearly the same as those in the CD2 hydrogels, which was consistent with the trend of cell survival measured by the CCK-8 assay. This finding suggests that the ECs embedded in hydrogels containing low concentration fullerenes may not generate of excess ROS to adversely affect cell viability in a 3D environment [45]. For long-term culture after 3 days, there was no significant difference in the proliferation rates of ECs among all the groups. This may be attributed that the CD2 hydrogel provides a favorable 3D growth environment, allowing healthy cells to proliferate [46]. However, at this stage, the interactions between cells and fullerenes may not be particularly pronounced, possibly due to the large spatial separation and lack of contact in the 3D environment. After culture for 7 days, the cell proliferation rate in the groups of CD2 hydrogels containing FNW-P25 (~320%) and FNW-P50 (~323%) were the highest among all groups and were significant different from that of the CD2 hydrogel control (~280%), as displayed in Figure 7(c). Meanwhile, the proliferation rates of ECs in the groups of CD2 hydrogels containing FNT-P25 (~278%) and FNT-P50 (~280%) demonstrated no significant difference from that of the control group. These results indicate that fullerenes of different nanoarchitectured structures affect the interaction of cells with the surrounding in a 3D environment.

### 3.8. Immunofluorescence staining and gene expression of ECs in the composite hydrogel

**Figure 8.**
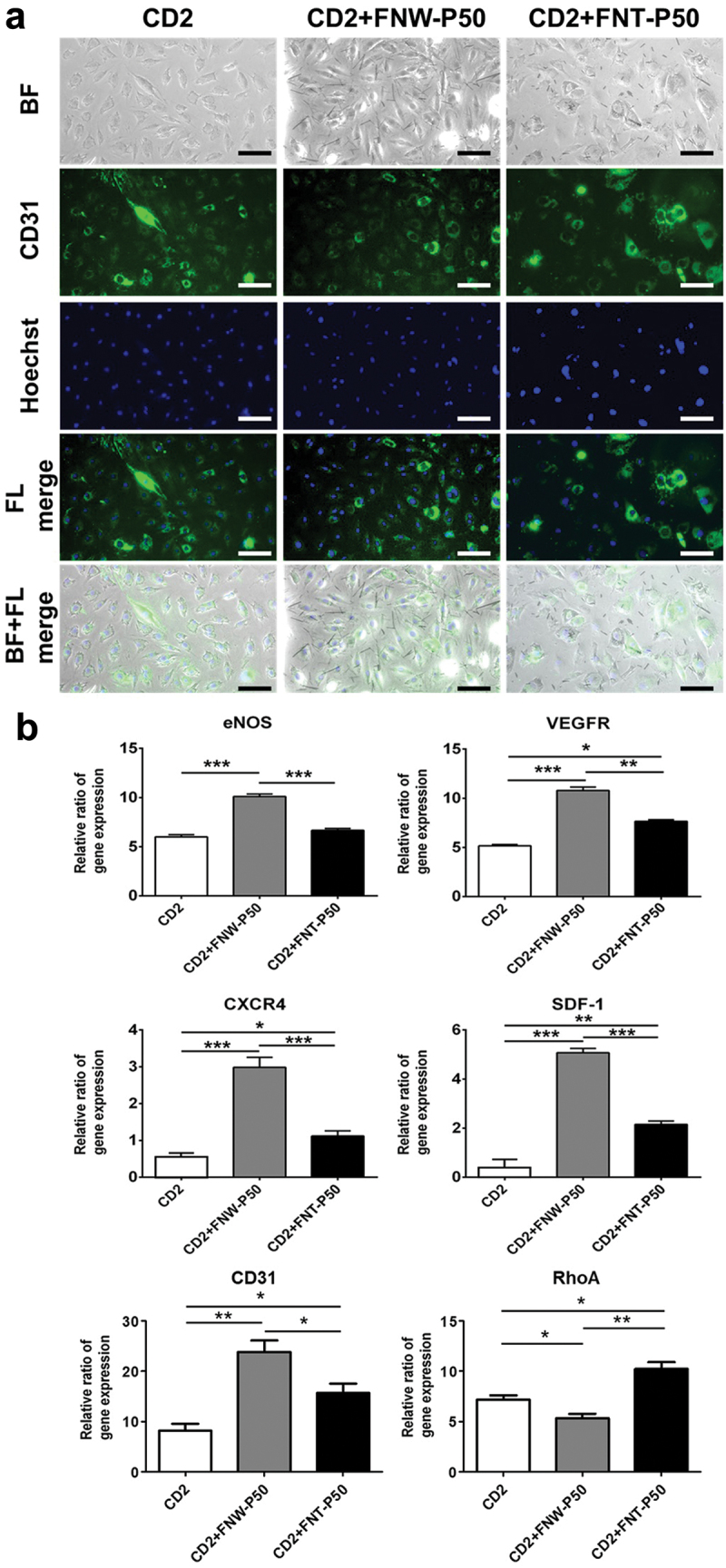
*In vitro* immunofluorescent staining and gene expression of ECs encapsulated in the composite CD hydrogels. (a) Immunofluorescent images of ECs stained for CD31 after culture in the composite CD hydrogels for 7 days. Scale bars:100 μm. ‘BF’ represents the bright field in short. ‘FL’ represents the fluorescent light in short. (b) Expression of vascular-related genes in ECs encapsulated in the composite CD hydrogels were analyzed by RT-PCR at 7 days. The expression levels were normalized to that of housekeeping gene (GAPDH) and represented by the relative ratios of gene expression. Asterisks indicate statistically significant differences, *p < 0.05, **p < 0.01, and ***p < 0.001.

Immunofluorescence staining for CD31 of ECs within the hydrogel containing FNW-P50 or FNT-P50 after culture for 7 days is shown in Figure 8(a). The ECs within the CD2 hydrogel containing FNW-P50 revealed elongated morphology and aligned more than those in the other groups. Meanwhile, ECs displayed rounded morphology and clustered distribution within CD2 hydrogel containing FNT-P50. In a 3D environment, variation of EC growth along nanomaterials leading further to the formation of vascular structures is associated with finely tuned biological and biophysical mechanisms [47]. Particularly, biomechanical interaction plays a pivotal role, with receptors on the EC surface engaging in specific interactions with the surrounding environment [48]. These interactions include both cell-cell adhesion and the dynamic interaction with the ECM, mutually guiding and orchestrating the directional growth of ECs. In the current study, our optimized self-healing hydrogel system exhibits excellent self-healing properties, which can promote biomechanical interactions between proliferated cells and between cells and the neighboring nanomaterials. Furthermore, the differences in morphology and growth of ECs may be attributed to the distinct aspect ratios between FNW-P and FNT-P. The FNW-P with a longer length (~64.7 µm) and higher aspect ratio (53.9) may facilitate focal adhesion of ECs through increased adsorption of ECM proteins to the long axes of FNW-P. This may advantageously influence cell spreading and orientation, promoting the alignment and growth of ECs along the arrangement of FNW-P.

Initially we considered that FNT-P with a smaller diameter and aspect ratio may be endocytosed by cells. However, the ECs did not exhibit a tendency to endocytose FNT-P in the current study. Instead, they formed clusters and adhered with FNT-P. This may be attributed to the relatively large size and low aspect ratio of FNT-P, resulting in inefficient fullerene-cell membrane interactions and subsequently affecting cellular uptake. In addition, the cavities at both extremes of FNT-P were small with length of ~5 µm and diameter of ~3.4 µm, preventing effective EC infiltration into the gaps [49]. Simultaneously, the dynamic regulation of the intracellular cytoskeleton stands out as a key determinant in the growth and morphology of ECs [50]. The orchestrated reorganization of microtubules and microfilaments, particularly during cell migration, is important to the complicate process of directing EC growth [51]. This dynamic cytoskeletal rearrangement serves as the structural foundation for the subsequent formation of vascular structures. Importantly, our optimized self-healing hydrogel system, with a mechanical strength suitable for EC growth (~400–500 Pa), facilitates cell migration and aggregation, as well as promotes the orchestrated formation of new blood vessels through enhanced intercellular interactions [52].

In addition to biomechanical influences, the role of growth factors and intercellular signaling is important for EC vascularization [53]. The elongation or cluster of ECs along fullerenes induced a finely tuned molecular response, causing substantial changes in gene expression pivotal for the formation of vascular structures, as demonstrated in Figure 8(b). This orchestrated process involves the modulation of key genes, including eNOS, VEGFR, SDF-1, CXCR4, CD31, and RhoA. The gene expression levels of eNOS, VEGFR, SDF-1, CXCR4, and CD31 in the group of CD2+FNW-P50 were significantly higher than those of the other groups. Meanwhile, the gene expression levels of VEGFR, SDF-1, CXCR4, and CD31 in the group of CD2+FNT-P50 were significantly higher than those of CD2 hydrogel control. In addition, the gene expression levels of RhoA in the group of CD2+FNW-P50 were significantly lower than those of the other groups. However, the gene expression levels of RhoA in the group of CD2+FNT-P50 were significantly higher than those in the other groups. RhoA, a member of the Rho GTPase family, plays a critical role in regulating the actin cytoskeleton [54,55]. The decreased RhoA activity in the group of CD2+FNW-P50 may result in the reduced formation of stress fibers, which are structures associated with cell contraction and stability. This reduction in contractility can lead to a more elongated morphology of ECs. The increased RhoA activity in the group of CD2+FNT-P50 typically results in a more contractile cell phenotype, leading to a rounder and more compact morphology. The gene expression level of eNOS did not differ significantly between the groups of CD2+FNT-P50 and the control CD2 hydrogel. eNOS is a central mediator of nitric oxide production in ECs, which regulates various physiological functions [56]. For ECs within CD2+FNW-P50, elongation-induced upregulation of eNOS expression can promote angiogenesis, regulate vasodilation, and enhance endothelial function, which supports EC survival and the establishment of a functional vascular network. For ECs within the CD2+FNW-P50 and CD2+FNW-P50, the upregulation of VEGFR in response to EC elongation and cluster enhances the cellular sensitivity to VEGF signaling, promoting EC proliferation and migration, which is significant to vascular morphogenesis [57]. Concomitantly, different EC morphogenesis is expected to modulate the expression of SDF-1 and its receptor CXCR4, critical elements in EC migration, stem cell homing, and angiogenesis [58]. The SDF-1/CXCR4 signaling pathway also affects the adhesion of ECs, promoting their attachment to the ECM [59]. Moreover, CD31 is a cell adhesion molecule primarily found on the surface of ECs and mediates cell-cell adhesion [60]. These changes in gene expression may influence EC recruitment, contributing to the dynamic process of vascular network formation.

In the broader intercellular regulatory, the Notch signaling pathway emerges as a central player that regulates development of elongated and adhesive cells, including ECs and NSCs [61]. These types of cells need directed migration, interactions, and adhesion for their physiological functions in the vascular and neural systems. Thus, their morphology and adhesion are crucial for adapting to their environments and fulfilling their physiological roles. In literature, EC elongation induces adjustments in the expression of Notch pathway components, influence cell development, and impact the delicate balance between tip and stalk cells, which is an essential aspect of angiogenesis [62]. The alteration of mechanical forces triggers activation of the Notch ligand DLL4, which is one of the most important signals in angiogenesis for ECs [63]. The incorporation of fullerenes in our optimized self-healing hydrogel system may provide mechanical boundary conditions as cues to affect the development of ECs and vascular tissue through Notch pathway. While Notch also plays a key role in neural development, an amino-functionalized fullerene tubes with a smaller size (1.5 µm long and 0.48 µm diameter) were endocytosed by neural stem cells to cause significant neural differentiation [15]. Interestingly, endocytosis by ECs was not apparent for either form of fullerene self-assemblies in the current study.

In summary, we developed chitosan-based self-healing hydrogels containing FNW-P or FNT-P as in vitro 3D platforms to investigate the impact of interactions between ECs and various nanoarchitectured structures of fullerenes in a 3D environment. The hydrogel developed for the system possesses a mechanical strength (~0.4 kPa) suitable for EC proliferation (~280% for culture after 7 days) and exhibits good self-healing properties, which facilitates cell migration and migration-induced morphological changes of cells. The proliferation rate of ECs reached a maximum of ~ 323% after culture for 7 days through the addition of limited concentration of FNW-P or FNT-P in the hydrogel system. Moreover, the morphology and vascular-related gene expression of ECs were significantly modulated because of the biomechanical influence of FNW-P and FNT-P with different aspect ratios possibly through intercellular regulatory mechanisms with growth factors. We believe that soft hydrogels and fullerenes provide unique environmental cues for the development of ECs and possibly affect the vascular formation. The combined use of self-assembled fullerenes and self-healing hydrogels may represent a novel approach to effectively control the morphology and arrangement of ECs in a 3D biomimetic microenvironment, which after further exploration could become a promising strategy for promoting vascularization in tissue engineering.

## 4. Conclusion

Two nanoarchitectured structures of self-assembled fullerenes (FNT and FNW) with different aspect ratios of 4.6 and 53.9 were synthesized from the dynamic and static LLIP methods, respectively. The aqueous stability of FNT-P and FNW-P increased after hydrophilic coating with Pluronic. After hydrophilic coating with Pluronic, the zeta potentials decreased from −6.5 ± 1.2 mV to −14.8 ± 2.1 mV for FNT-P and from −15.2 ± 2.4 mV to −34.2 ± 1.8 mV for FNW-P, enhancing the aqueous stability of self-assembled fullerenes. For 2D culture with self-assembled fullerenes after 7 days, the ECs significantly proliferated ~4-fold in the presence of low concentration FNT-P50 or FNW-P50. A rather soft chitosan-based self-healing hydrogel with G’ of ~ 0.4 kPa was also developed in this study to disperse FNW-P or FNT-P. The composite hydrogel systems provide a suitable 3D environment for proliferation, migration, and morphological change of ECs through the biomechanical influence of FNW-P and FNT-P with different aspect ratios and intercellular regulatory mechanisms with growth factors. The proliferation rate of ECs reached ~323% and ~280% after culture for 7 days through the addition of FNW-P50 or FNT-P50 in the hydrogel system. The ECs in the hydrogel systems containing FNW-P50 exhibited an elongated morphology and grew in alignment with the arrangement of the FNW-P. In contrast, the ECs in the hydrogel systems containing FNT-P50 displayed a rounded morphology and clustered distribution with FNT-P. The ECs did not exhibit a tendency to endocytose either FNW-P or FNT-P. Instead, they appeared to adhere and formed elongate alignment or clustered distribution with the arrangement of FNW-P or FNT-P. Moreover, the vascular-related gene expressions of ECs significantly upregulated through the biomechanical and biological interaction between fullerenes and ECs. We believe that soft hydrogels and fullerenes offer distinct environmental cues influencing EC development and potentially impacting vascular formation. The combined use of self-assembled fullerenes and self-healing hydrogels may present an innovative method to precisely manipulate EC morphology and arrangement in a 3D biomimetic microenvironment. Following further investigation, this approach holds promise as a strategy for advancing vascularization in tissue engineering.

## Supplementary Material

Supplemental Material

Supplemental Material
