# Supplementary material for: Interaction of vascular endothelial cells with hydrophilic fullerene nanoarchitectured structures in 2D and 3D environments
Source: Sci Technol Adv Mater. 2024 Feb 13;25(1):2315014. doi: 10.1080/14686996.2024.2315014 (PMC10901190; doi:10.1080/14686996.2024.2315014)
Supplement: Supplemental Material [file TSTA_A_2315014_SM3271.docx]

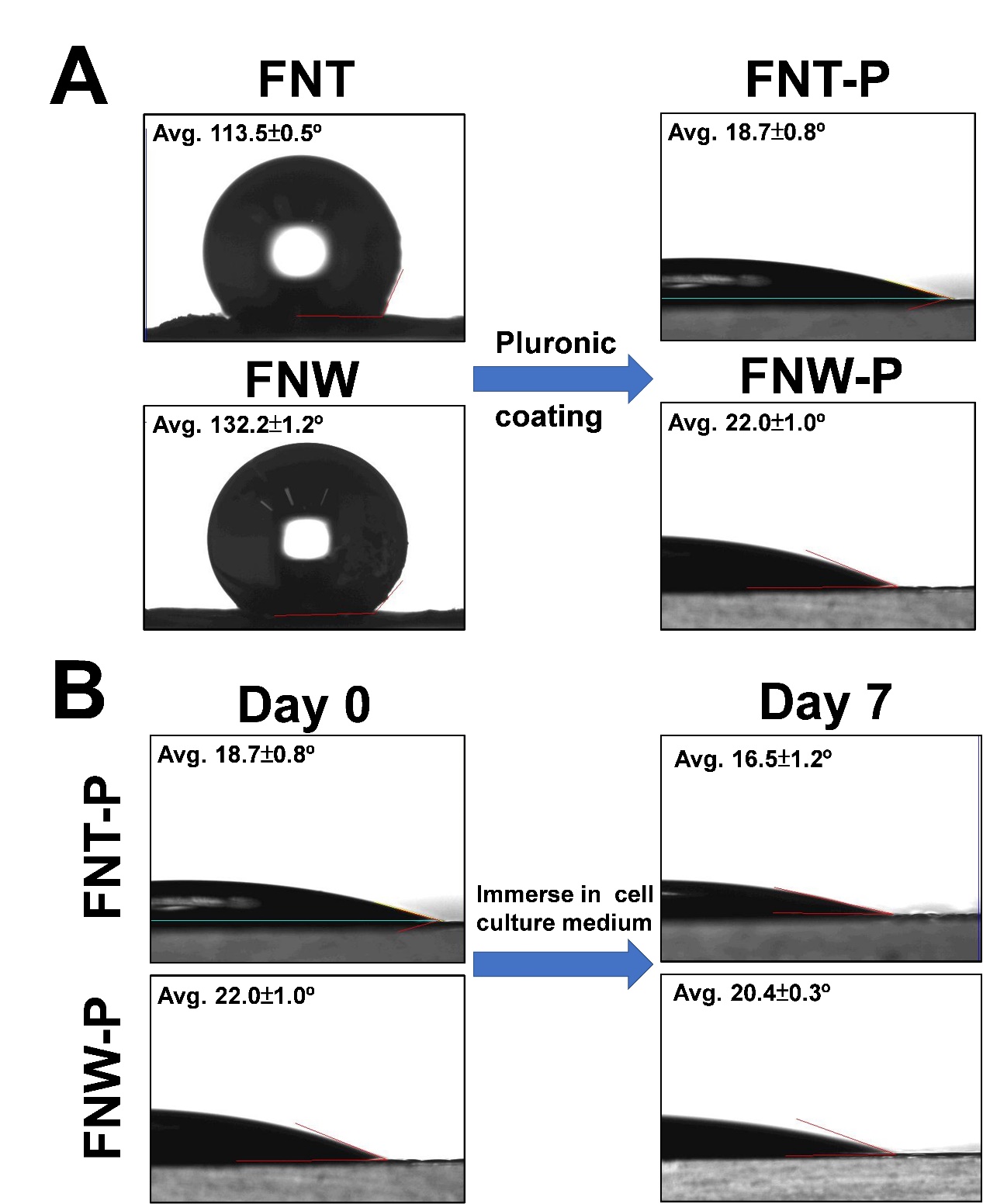


Figure S1. Contact angle measurement of self-assembled fullerenes. (A) The change in contact angle of self-assembled fullerenes before and after hydrophilic surface modification by Pluronic. (B) Contact angle measurement of hydrophilic self-assembled fullerenes (FNT-P and FNW-P) after immersion in cell culture medium for 7 days.


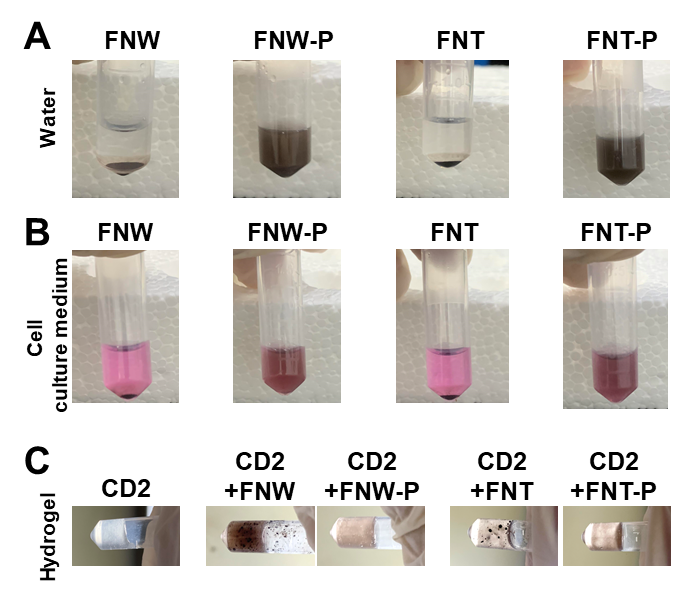


**Figure S2.** Images of the Pluronic-coated self-assembled fullerenes and non-coated self-assembled fullerenes in (A) water, (B) cell culture medium, and (C) CD2 hydrogel.
